# An Account of Two Cases of Pemphigus; to Which Is Added a Fact Relative to the Early Practice of Inoculation of the Small Pox in Wales

**Published:** 1790

**Authors:** John Ring

**Affiliations:** Surgeon in London


IV. *An Account of two Caſes of Pemphigus; to which is added a Fact relative to the early Practice of Inoculation of the Small Pox in Wales. Communicated in a Letter to Dr.* Simmons *by Mr.* John Ring, *Surgeon in London.*

HAVING ſeen ſome obſervations on pemphigus in the London Medical Journal, (Vol. X.) I take the liberty of communicating to you a ſhort account of two inſtances of that diſeaſe which have occurred in my practice, and in both of which it appeared in a very mild form, and was cured without difficulty.

The ſubject of the firſt of theſe caſes was a child of Mr. Hawkins, at No. 86, Oxford Street, who had an eruption of very large veſicles of an oval ſhape, and in number about fourteen, which appeared almoſt entirely on the fore part of the cheſt, on the third and fourth day day after his birth. No material indifpofition attended this eruption. The veficles foon burft, from the preffure neceffarily made on that part by his clothes. The parts, being dreffed with ung. ceræ albæ, foon healed. No internal medicine was given but a little magnefia occafionally to obviate coftivenefs.

The fecond patient was Mr. Grant, of Mill Street, Hanover Square, who had an eruption of a veficle of the fize of a large nutmeg on his leg, near the middle of the tibia, which was followed within twenty-four hours by a fimilar one on the fame part of the other leg. Slight febrile fymptoms attended the eruption, for which fuitable medicines were given.

I advifed him not to open the veficles, notwithftanding they were rather painful; but after fome days one of them broke accidentally; and as no ill confequence followed, I punctured the other at its bafe. The puncture being fmall, clofed, and the blifter half filled again by the next day. The fluid was again difcharged, and the patient was freed from pain. The parts affected foon healed.

If it appears from frequent experiments that the veficles may be opened with fafety, and a mild ointment applied, much eafe may be afforded to the patient, and by leſſening the irritation of the ſkin the fever, and conſequently the danger attending the caſe, will be diminiſhed.

It has lately been obſerved in the London Medical Journal *, that in ſome parts of Africa a cuſtom prevails of buying the ſmall pox. It may not be improper to add, that a ſimilar cuſtom prevailed in South Wales previouſly to the introduction of inoculation by Lady Mary Wortley Montague; or, rather, the ſmall pox was communicated in a mode ſimilar to the preſent, but under another name. The perſon who wiſhed to have the diſorder bargained with another who had it for ſo many puſtules at a certain price, and rubbed the matter on the ſkin, either ſound or previouſly wounded, or punctured it with an inſtrument firſt infected. From the moſt credible teſtimonies it appears that this practice was common in many parts of Wales from time immemorial, but not in the hands of medical practitioners as at preſent.—Vide Philoſ. Tranſ. No. 375, p. 262 and 267.

*Swallow Street,*

July 20, 1790.

* See page 141 of this volume.

